# Analysis of the Expression of LSF Transcription Factor in the Regulation of Transcription and TSG101 during the Neoplastic Transformation of Endometrial Cells

**DOI:** 10.3390/cells13070580

**Published:** 2024-03-26

**Authors:** Rafał Ziemiński, Aleksandra Stupak, Maciej Kwiatek, Tomasz Gęca, Alicja Warowicka, Karolina Hejne, Anna Kwaśniewska, Anna Goździcka-Józefiak, Wojciech Kwaśniewski

**Affiliations:** 1 Department of Obstetrics and Pathology of Pregnancy, Medical University of Lublin, 20-059 Lublin, Poland; rafal.zieminski@gmail.com (R.Z.); maciej.kwiatek@umlub.pl (M.K.); tomasz.geca@umlub.pl (T.G.); anna.kwasniewska@umlub.pl (A.K.); 2 Department of Molecular Virology, Institute of Experimental Biology, Adam Mickiewicz University in Poznan, 61-712 Poznań, Poland; alicja@amu.edu.pl (A.W.);; 3 Department of Pathomorphology and Forensic Medicine, School of Medicine, Collegium Medicum, University of Warmia and Mazury, 11-082 Olsztyn, Poland; 4 Department of Gynecology Oncology and Gynecology, Medical University of Lublin, 20-059 Lublin, Poland

**Keywords:** carcinogenesis, LSF transcription factor, TSG101, endometrial cancer

## Abstract

Previous research indicates that carcinogenesis involves disrupting the functions of numerous genes, including factors involved in the regulation of transcription and cell proliferation. For these reasons, in endometrial carcinogenesis, we decided to investigate the expression of TSG101 (a suppressor of tumor transformation) and LSF (a transcription factor involved in numerous cellular processes, such as cell cycle regulation, cell growth, development, and apoptosis). LSF may be involved in the regulation of TSG101 expression. The research material consisted of endometrial cancer samples from 60 patients. The control group consisted of normal endometrium samples donated by 60 women undergoing surgery for benign diseases of the female reproductive organs. The samples were subjected to immunohistochemical staining with antibodies specific to TSG101 and LSF. Specific antibodies were used to identify TSG101 and LSF in the examined histopathological preparations. An approximately 14-fold lower risk of endometrial cancer development was observed in patients with TSG expression in more than 75% of the assessed cells (4% vs. 36%; OR = 0.07; *p* = 0.0182). There was a four-fold lower risk of endometrial cancer development in patients with LSF expression in more than 50% of the assessed cells (32% vs. 64%; OR = 0.26; *p* = 0.0262). A more than three-fold lower risk of endometrial cancer development was observed in patients with LSF expression in more than 75% of the assessed cells (24% vs. 52%; OR = 0.29; *p* = 0.0454). Endometrial cancer was diagnosed in those with a lower level of TSG101 expression than in those with a cancer-free endometrium. Decreased expression of TSG101 may be a marker of endometrial cancer, and increased expression of LSF when diagnosed with endometrial cancer may indicate greater advancement of the disease. These markers might be used as diagnostic and prognostic markers—however, there is a lack of a correlation between them.

## 1. Introduction

### 1.1. Carcinogenesis and Molecular Biology

Previous research indicates that, in carcinogenesis, the activity of many genes is disrupted, especially those involved in the processes of angiogenesis, adhesion, proliferation, immune response, proteolysis, differentiation, and cell death [1]. Despite the spectacular contribution that molecular biology has made in the field of carcinogenesis over the last half-century, mortality due to cancer declined by almost 30% [2].

Endometrial cancer is the sixth most common cancer in the world, as well as the most common and least aggressive malignant tumor of the female reproductive tract, with a five-year survival rate of up to 80% [3]. It is estimated that 320,000 new cases of this cancer are diagnosed each year. Women living in highly developed countries are more likely to develop endometrial cancer (5.9%) compared to countries with low resources (4.0%), although the mortality rate is higher in the latter. The accumulative risk of endometrial cancer before age 75 is estimated to be 1.6% in high-income regions and 0.7% in low-income countries. This may be related to high rates of obesity and physical inactivity, two major risk factors, in high-income countries [4].

In Poland, in the years 2010–2020, endometrial cancer was the fourth most common cancer in women; 5119 cases were registered (standardized coefficient of 54.71/100 thousand), and 1797 deaths were reported (standardized coefficient of 19.21/100 thousand) [5]. Endometrial cancer is the twelfth leading cause of cancer death in women, and the number of deaths has remained stable despite the incidence almost doubling. This trend, which has been observed for over two decades, will most likely continue. Most cases occur among women over the age of fifty [3].

Recent studies have shown that neither the traditional histopathological classification nor its division into two types according to Bokhman allows for a reliable assessment of the prognosis and response to treatment of endometrial cancer [6]. However, this information may be obtained through the molecular classification introduced in 2013 as the Cancer Genome Atlas (TCGA) from the United States National Cancer Institute (NCI) and the National Human Genome Research Institute [7]. This classification identifies four molecular subtypes of endometrial cancer, differing in the types of DNA mutation, immunogenicity, and prognosis, and requiring different therapeutic approaches.

The introduction of this molecular classification motivated a search for additional molecular prognostic factors in addition to the previously known ones, such as a high S phase fraction, aneuploidy, PTEN absence, PIK3CA mutation, p53 mutation, and Her-2/neu overexpression [8].

### 1.2. Gen TSG101

One of the factors that may be involved in carcinogenesis is TSG101. The TSG101 gene is located on chromosome 11.p15.1–p15.2, in a region commonly involved in the loss of heterozygosity. The TSG101 protein is involved in a variety of biological processes, such as ubiquitination, transcriptional regulation, endosomal transport, virus budding, cell proliferation, and survival. It is suggested that TSG101 is an important factor in maintaining cellular homeostasis and that disturbances in its function lead to cancer transformation.

For this reason, TSG101 has long been considered a tumor suppressor factor responsible for genetic stability and regulation of the cell cycle, and its mutations may lead to the disruption of these processes and, as a result, cancer development [9]. In reality, however, its role is not as clear as it may seem. In 1999, Chang et al., based on a review of available studies, found that patients with a loss of heterozygosity on the short arm of chromosome 11, where the TSG101 gene is located, were more likely to develop cervical and breast cancer. TSG101 expression is a constitutive element in the cells of many human tissues. Increased TSG101 expression was found in papillary thyroid cancers and tumors of the breast, ovary, and gastrointestinal tract, while its reduced expression was observed in cervical and endometrial cancers [10].

Recently, there has been a lot of research in cancer diagnostics on the use of exosomes as biomarkers for cancer detection. TSG101 is one of the primary proteins detected in exosomes [11,12,13,14,15].

### 1.3. LSF Transcription Factor

LSF belongs to the TFCP2/Grainyhead family of transcription factors. These factors are present in both animals and fungi. This family is divided into two separate subfamilies, one of which includes Grainyhead-like proteins 1–3 (GRLH 1–3) and another which includes LSF (synonyms: CP2, TFCP2, LBP-1c), TFCP2L1 (synonyms: CRTR-1, LBP-9), and UBP1 (synonyms: LBP-1a and NF2d9). The DNA-binding domain of these proteins contains a characteristic immunoglobulin-analogous structure, similar to that of the widely known TP53 tumor suppressor gene.

LSF was initially detected by its ability to activate the primary promoter of simian virus SV40. The SV40 factor (LSF) is variably called CP2 or LSF and is encoded by the TFCP2 gene located on the long arm of chromosome 12. Human LSF is a protein composed of 502 amino acids with a molecular weight of approximately 57 kDa. Transcription factors from the LSF/TFCP2L1/UBP1 subfamily are involved in various stages of cancer development. LSF promotes the development of hepatocellular carcinoma, pancreatic cancer, and breast cancer, and may also be important in the development of cervical, colon, and oral cancer. However, LSF can also act in the opposite direction as a tumor suppressor, such as in melanoma. Moreover, LSF is involved in epithelial–mesenchymal transition and enhances angiogenesis [16]. Deregulated LSF expression can facilitate entry into the G1/S phase of the cell cycle, promote DNA synthesis, stimulate transformation, and facilitate cancer cell survival. LSF inhibition causes apoptosis during the S phase or cell cycle arrest at the G1/S transition.

The attempt to link the activity of TSG101 and LSF is a relatively new concept, and there are few scientific works on this subject. The first group to note a link was Broniarczyk et al., who found an LSF-binding site in the promoter of the TSG101 gene during their research on the mechanism of carcinogenesis in cervical cancer in 2014 [10]. To identify the mechanisms responsible for the downregulation of TSG101 in cervical carcinogenesis the TSG101 promoter was analyzed with the usage of cis-element cluster finder software (Zlab gene regulation tools, Boston Univeristy, Boston, MA, USA). The analysis took into account the location and quantity of LSF binding sites. Fourteen binding sites for LSF were revealed on TSG-101 promoter. This suggests that the impact of LSF for TSG-101 transcription can be significant, and both can play role in the carcinogenesis.

## 2. Objective

The aim of this study was to assess the expression of the transcription factor LSF and TSG101 during the neoplastic transformation of endometrial cells and to answer the question of whether the analyses of the expression of TSG101 and LSF could have practical applications as prognostic factors in patients with endometrial cancer.

## 3. Materials

The analysis included samples donated by 120 patients operated on at the Department of Gynecological Oncology and Gynecology of the Medical University of Lublin, 2015–2020.

The tests and procedures performed were in accordance with the requirements of the Helsinki Declaration of 1975, as amended in 2000. Each patient signed an informed consent form to participate in the study. The research performed was approved by the Bioethics Committee of the Medical University of Lublin (number: KE-0254/151/2015).

Patients with the following postoperative histopathological diagnoses were qualified for the study:

- Study group: 60 women diagnosed with endometrial cancer;
- Control group: 60 women with benign diseases of the female reproductive organs.

The test materials used to evaluate TSG101 and LSF were paraffin-embedded tissue sections of the endometrium (Sigma Aldrich, St. Louis, MO, USA) collected from the patients during surgery.

The criterion for including patients in the study group was a postoperative histopathological diagnosis of endometrial cancer.

The criteria for including patients in the control group were patients in the perimenopausal period without malignant gynecological diseases.

The criteria for excluding patients from the study were as follows: use of hormone replacement therapy, other malignant tumors of the female genital organs, systemic diseases, ischemic heart disease or previous heart attack, peripheral vascular diseases, thyroid diseases and other endocrine diseases, and/or diseases of the liver and biliary tract.

During the immunohistochemical assays, samples from 10 patients each from the study and control groups were rejected due to unsatisfactory histopathological results and damage to the specimens. Ultimately, 50 samples each from the study and control groups were selected for testing.

In the group of 50 endometrial adenocarcinomas, a high degree of differentiation (G1) was found in 14/50 (28%) patients, a moderate degree (G2) was found in 24/50 (48%), and a low degree (G3) was found in 12/50 (24%). For the purpose of clinical analysis, the staging of endometrial cancer according to the FIGO classification was performed [17]. A total of 16 patients had stage IA (16/50; 32%), while 26 patients had stage IB (26/50; 52%). Patients with stage II (2/50; 4%), stage IIIB (4/50; 8%) and stage IIIC (2/50;4%) were also diagnosed. Penetration of the uterine muscular membrane was defined as being less than halfway in 40 patients, with deep infiltration penetrating more than halfway through the uterine wall in 20 patients. The mean ± std age of the patients in the control group was 63 ± 11.1, while in the study group, it was 67 ± 10.5.

## 4. Methodology

### 4.1. Methodology of Immunohistochemical Tests

The preparation of the biological material and immunohistochemical staining were carried out at the Pathomorphology Laboratory of the University Clinical Hospital No. 1 in Lublin.

Tissue sections, in accordance with the standard procedures at the Pathology Laboratory, were fixed in formalin, rinsed in distilled water, and then placed in an automatic Leica carousel Tissue Processor Microsystem TP1020 (Leica, Wetzlar, Germany), in which the process of dehydration in alcohol with increasing concentrations was carried out, followed by three-fold dehydration in acetone, clarification in xylene, and infiltration with liquid paraffin.

The following reagents were used in this step: EtOH acetone line, Cat. No. M00058238 (POCH SA, Gliwice, Poland); ethyl alcohol anhydrous, 99.8% PURE, Cat. No. 396480111; acetone CZDA, Cat. no. 102480111; xylene CZDA, Cat. No. 520860119. Histopathological paraffin premium with DMSO (Pathosolutions, ELEKTROMED) was used to embed the biological material, Cat. No. EM-851001 (Niepołomice, Poland).

After cooling, the obtained blocks were cut on a semi-automatic Leica rotary microtome, model RM 2245 (Leica Biosystems Nussloch GmbH, Wetzlar, Germany), into sections 3.5–4 μm thick. The blades used for cutting were the Sakura Accu-Edge Low-Profile Microtome Blades Stainless Steel, Cat. No. 4686 (Feather Safety Razor Co., Ltd., Osaka, Japan). The cut sections were then placed on the surface of water in a Bio-Optica (Bio-Optica, Milano, Italy) water bath. The paraffin sections prepared in this way were placed on silanized Super Frost Plus-MENZEL GLASSER glass slides, Cat. No. J1800AMNZ, (Menzel-Gläser, Wetzlar, Germany). The obtained preparations were stored in a refrigerator at minus 8 °C until IHC staining was performed.

### 4.2. Preparation Staining Protocol

Immediately before immunohistochemical staining, the microscope slides were placed on a Bio-Optica (Milano, Italy) heating plate set at 60 °C for 60 min to permanently glue the sections to the glass slides. Then, the standard dewaxing and hydration process was carried out.

In the next stage, the sections were subjected to a thermal unmasking procedure of antigenic determinants in an MLL 547 water bath (AJL Electronic, Kraków, Poland), placing them for 20 min in the unmasking solution (Tris-based, high pH) at a temperature of 95–99 °C, Cat. No. H-3301-250, (Vector Laboratories, Newark, CA, USA).

The activity of endogenous tissue peroxidases was blocked by incubating the sections in a 3% hydrogen peroxide (H_2_O_2_) solution for 5 min (Hasco-Lek SA, Wrocław, Poland). After this time, the preparations were rinsed twice in PBS buffer for 5 min. The slides were dried and wiped as in the previous step. The sections were then incubated in blocking serum provided by the kit manufacturer (ready-to-use 2.5% normal horse serum).

After 20 min, the primary polyclonal antibody directed against the tested human antigen was applied, suspended in an appropriate diluent, for 30 min.

After another three washes in PBS buffer, the preparations were incubated for 30 min with a species-specific immunoglobulin solution, appropriate for the primary antibody used.

The slides were washed as in the previous steps. A color reaction was obtained by incubating the sections in a peroxidase substrate solution for the time necessary to achieve the appropriate level of color intensity (DAB peroxidase substrate, Cat. No. SK-4100, Vector Laboratories, CA, USA) (approx. 5 min).

After rinsing the slides in distilled water (twice for 5 min), counterstaining was performed. The cell nuclei were stained with hematoxylin according to Mayer (Cat. No. 468860448, POCH SA, Gliwice, Poland). In the final step, the slides were dehydrated through the previously described alcohol–acetone dehydration series, exposed to xylene, and mounted in medium (Shandon Consul-Mount^TM^ Histology Formulation, Cat. No. 9990440, Thermo Scientific, Waltham, MA, USA).

### 4.3. Detection of TSG101 and LSF antigens

The TSG101 and LSF antigens in the examined histopathological preparations were detected using the BenchMark^®^ GX Ventana apparatus (Medical Systems, Inc., Roche, Arizona, USA).

The following antibodies were used:

- Anti-LSF Purified Monoclonal Mouse IgG1 (clone: 14/LSF) (1:50 dilution) (BD Transduction Laboratories^TM^, Cat. No. 610818, BD Biosciences, North Brunswick Township, NJ, USA);
- TSG101 (C-2): sc-7964 Mouse Monoclonal Antibody (1:50 dilution) (Santa Cruz Biotechnology, Inc., Dallas, TX, USA).

Ventana antibody reagent was used to dilute the antibodies (Diluent, Cat. No. 251-018, Roche, Tucson, AZ, USA).

The entire process, starting from the histopathological preparation at the stage of deparaffinization and exposure of the antigens, was carried out automatically in a device managed by a computer with NexES software containing optimized staining protocols. The final process of dehydration, rinsing in xylene, and sealing in mounting medium was performed manually.

Ventana reagents were used in accordance with the manufacturer’s recommendations (Medical Systems, Inc., Roche, Arizona, USA): reaction buffer concentrate (10×), Cat. No. 950-300; EZ Prep concentrate (10×) solution, Cat. No. 950-102; Cell Conditioning 1 (CC1), Cat. No. 950-124; Liquid Coverslip (High temp., Predilute, LCS), Cat. No. 650-010; Ultra View Universal DAB Detection Kit, Cat. No. 760-500; reaction buffer (10×), Cat. No. 950-300; bluing reagent, Cat. No. 760-2037; hematoxylin II, Cat. No. 790-2208; Prep Kit, Cat. No. 1637700. Dispensers were used to deliver the antibodies to the Ventana series slide-staining machine.

### 4.4. Immunohistochemical Analysis of TSG101 and LSF Expression

The following antibodies were used: (i) Anti-LSF Purified Monoclonal Mouse IgG1 (Clone: 14/LSF) (1:50 dilution) (BD Transduction Laboratories ^TM,^ Cat. No. 610818, BD Biosciences, North Brunswick Township, NJ, USA); (ii) TSG101 (C-2): sc-7964 Mouse Monoclonal Antibody (1:50 dilution) (Santa Cruz Biotechnology, Inc., Dallas, TX, USA).

Ventana antibody reagent was used to dilute the antibodies (Diluent, Cat. No. 251-018, Roche, Tucson, AZ, USA).

The final process of dewatering and rinsing in xylene and sealing in the medium was performed manually. As recommended, the authors used the following reagents from Ventana Medical Systems, Inc. (Roche, AZ, USA): reaction buffer concentrate (10×), Cat. No. 950-300; EZ Prep concentrate (10×) solution, Cat. No. 950-102; Cell Conditioning 1 (CC1), Cat. No. 950-124; Liquid Coverslip (high-temp., Predilute, LCS), Cat. No. 650-010; Ultra View Universal DAB Detection Kit, Cat. No. 760-500; reaction buffer (10×), Cat. No. 950-300; bluing reagent, Cat. No. 760-2037; hematoxylin II, Cat. No. 790-2208; Prep Kit, Cat. No. 1637700. Dispensers were used to deliver the antibodies to the Ventana series slide-staining machine.

### 4.5. Qualitative Assessment of TSG101 and LSF Expression

The immunohistochemical assessment of protein expression was performed independently by two pathologists blinded to the patient’s final diagnosis (University of Warmia and Mazury, Olsztyn, Poland). The permanent preparations were viewed using an Olympus BX45 (Tokyo, Japan) light microscope with the 5×, 10×, 20×, and 40× objective lenses (×10 in the eyepiece), and photographs were taken on an Olympus BX53 light microscope with an Olympus SC50 camera using Olympus cellSens Entry software V2.3. The estimated percentages of cells positive for TSG101 and LSF expression were assessed according to the following:

- IS (intensity score): no color reaction (none), weak reaction (weak), and medium reaction (moderate); a strong reaction was not present in any of the preparations;
- Quantity: ranges of 0%, <25% stained cancer cells/normal endometrium, 25–50%, 50–75%, >75%.

The evaluation technique relied on the examination of the color density and the distribution of colors. The percentage of positive cells was calculated by dividing the number of immunopositive cells by the total number of cells, based on the variables. A minimum of 5000 cells were enumerated for each of the groups under analysis. Below are photos of some of the analyzed preparations.

### 4.6. Statistical Analysis

The data were collected in an Excel spreadsheet (Microsoft Office Suite) and subjected to statistical analysis using *Statistica* (version 13 PL) and MedCalc (version 15.8 PL) software. For the descriptive characteristics of the quantitative variables, the following measures of clustering were used: mean, median, and measures of dispersion, such as the standard deviation, interquartile range, minimum, and maximum. The normality of the distribution of the studied variables was assessed using the D’Agostino–Pearson test. In cases of quantitative data with a normal distribution, parametric tests were used (Student’s *t* test for comparisons of independent quantitative variables, and Pearson’s test for correlations). However, if the data distribution did not comply with the Gaussian curve (was different from the normal), non-parametric tests were used (Mann–Whitney U test for comparisons of quantitative independent variables, the Spearman test for correlations). Regardless of the data distribution, categorical variables (more than two compared groups) were compared using the chi-squared test, while dichotomized variables were compared using Fisher’s exact test (maximum of two compared groups). The risk of an unfavorable event was estimated using the odds ratio test. Statistically significant and selected non-significant results were graphically presented using bar charts (for comparisons of the frequency distribution of categorical variables in the risk assessment), box-and-whiskers (for comparisons of the distribution of quantitative variables), scatter charts (for correlations), or ROC curves (in the assessment of the usefulness of the diagnostic tests of the tested markers). In all analyses, results for which the *p* values were less than 0.05 (assumed inference error 5%) were considered statistically significant.

## 5. Results

**Figure 1:**
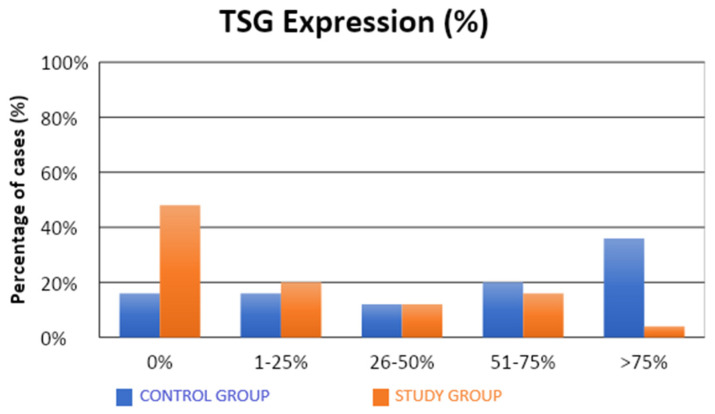
Comparison of TSG expression (%) in the control and study groups.

**Figure 2:**
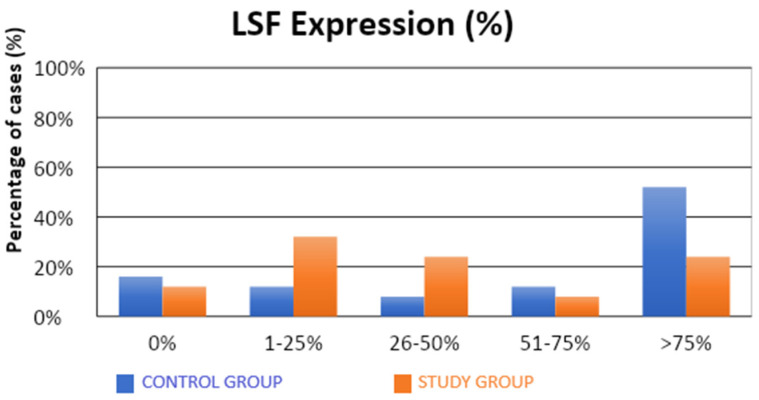
Comparison of LSF expression (%) in the control and study groups.

The characteristics of the demographic, clinical, and molecular variables of the control and study groups are presented in Table 1. Significantly fewer samples with high TSG expression were observed in the study group compared to the control group (4% vs. 36%; *p* = 0.0312; Table 1, Figure 1). There were no statistical differences between the groups in terms of LSF expression (Table 1, Figure 2). Our results suggest that high level of expression of TSG-101 is rare in endometrial cancer, and in most cases of EC its expression is low.

**Figure 3:**
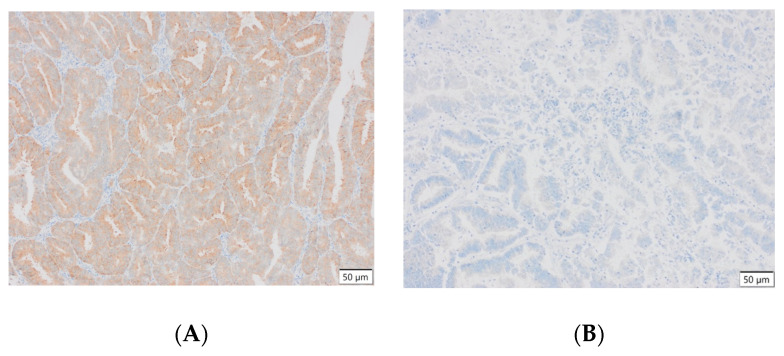

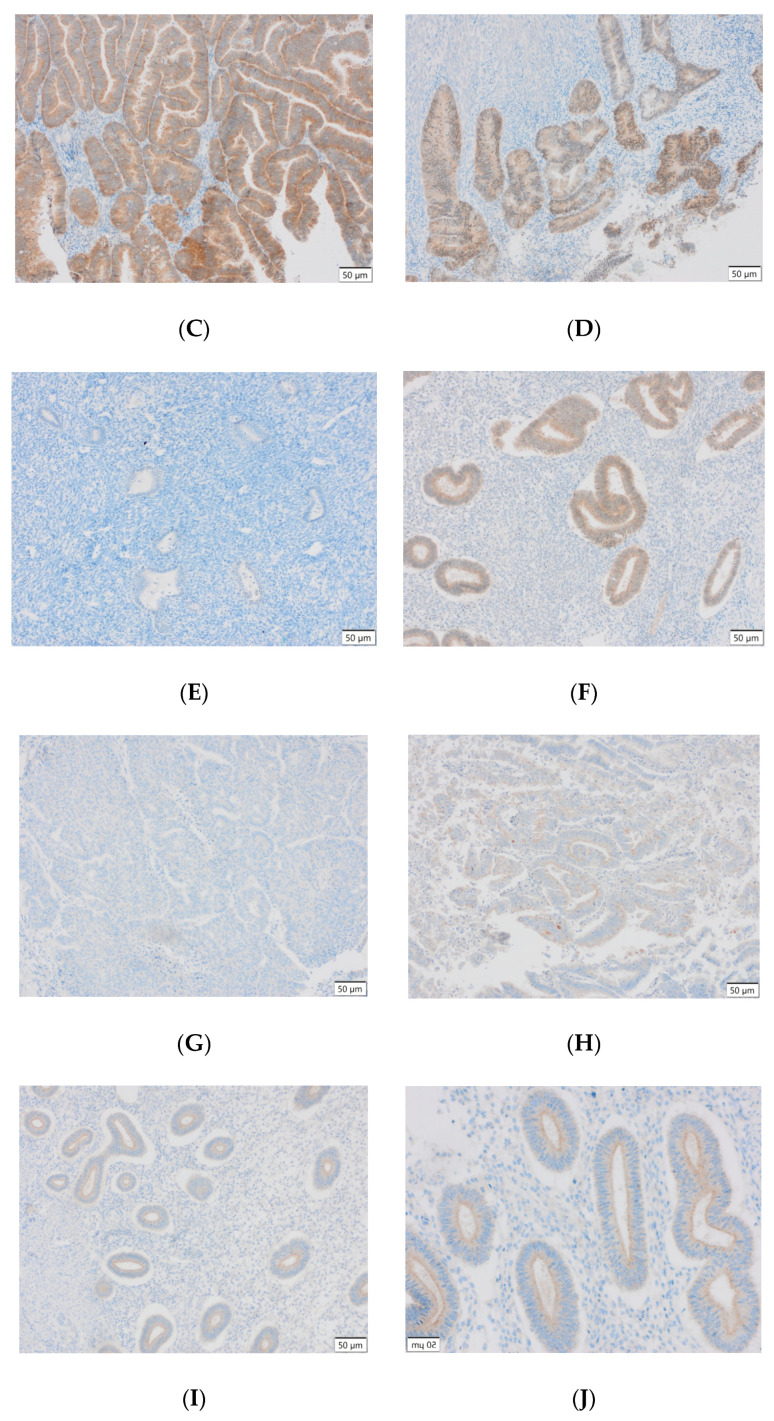

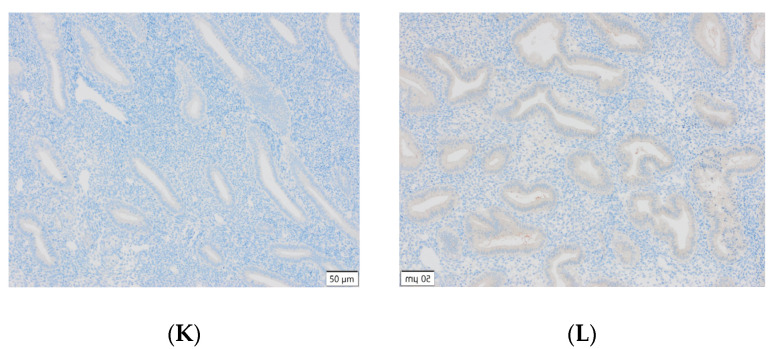
(**A**) Endometrial carcinoma, LSF; IS: moderate; quantity > 75%. (**B**) Endometrial cancer, LSF BDTL, San Jose; IS: none; quantity: 0%. (**C**) Endometrial cancer, LSF BDTL, San Jose; IS: moderate; quantity: 25–50%. (**D**) Endometrial cancer, LSF BDTL, San Jose; IS: weak; quantity > 75%. (**E**) Physiological endometrium, LSF BDTL, San Jose; IS: none; quantity: 0%. (**F**) Physiological endometrium, LSF BDTL, San Jose; IS: moderate; quantity > 75%. (**G**) Endometrial cancer, TSG100 (CAB004283), Santa Cruz; IS: none; quantity: 0%. (**H**) Endometrial cancer, TSG100 (CAB004283), Santa Cruz; IS: weak; quantity > 75%. (**I**) Physiological endometrium, TSG100 (CAB004283), Santa Cruz; IS: moderate; quantity > 75%. (**J**) Physiological endometrium, TSG (CAB004283), Santa Cruz; IS: moderate; quantity > 75%. (**K**) Physiological endometrium, TSG (CAB004283), Santa Cruz; IS: none; quantity: 0%. (**L**) Physiological endometrium, TSG (CAB004283), Santa Cruz; IS: weak; quantity > 75%.

Representative results of the immunohistochemical tests performed are presented in the photographs below (Figure 3).

All examined patients were diagnosed with endometrial cancer, glandular type, based on the histopathological examination. Details regarding the FIGO classification, nodal status, and histopathological grade are presented in Table 2.

### 5.1. Assessment of the Risk of Developing Endometrial Cancer Depending on the Presence of Specific Variables

About 50% of patients with endometrial cancer had hypertension versus 10% of the control group. In the case of all other analyzed demographic and clinical variables, there was no significant impact on the risk of endometrial cancer development.

A significantly lower (approximately five-fold) risk of endometrial cancer development was observed in patients with TSG expression in more than 1% of the assessed cells (52% vs. 84%; OR = 0.21; *p* = 0.0197). Similarly, there was a significantly lower (approximately five-fold) risk of developing endometrial cancer in patients with TSG expression in more than 25% of the assessed cells (32% vs. 68%; OR = 0.22; *p* = 0.0129). There was a significantly lower (also approximately five-fold) risk of developing endometrial cancer in patients with TSG expression observed in more than 50% of the assessed cells (20% vs. 56%; OR = 0.20; *p* = 0.0113). A significantly lower (approximately 14-fold) risk of endometrial cancer development was observed in patients with TSG expression in more than 75% of the assessed cells (4% vs. 36%; OR = 0.07; *p* = 0.0182).

The observed expression of LSF in a proportion above 1% or 25% of the evaluated cells did not have a statistically significant impact on the susceptibility to endometrial cancer. There was a significantly lower (also approximately four-fold) risk of developing endometrial cancer in patients in whom LSF expression was observed in more than 50% of the assessed cells (32% vs. 64%; OR = 0.26; *p* = 0.0262). A significantly lower (over three-fold) risk of endometrial cancer development was observed in patients with LSF expression in more than 75% of the assessed cells (24% vs. 52%; OR = 0.29; *p* = 0.0454).

Detailed data presenting the risk assessment of endometrial cancer depending on the occurrence of specific molecular changes are presented in Table 3.

### 5.2. Assessment of Correlations among Selected Markres in the Control and Study Groups

**Figure 4:**
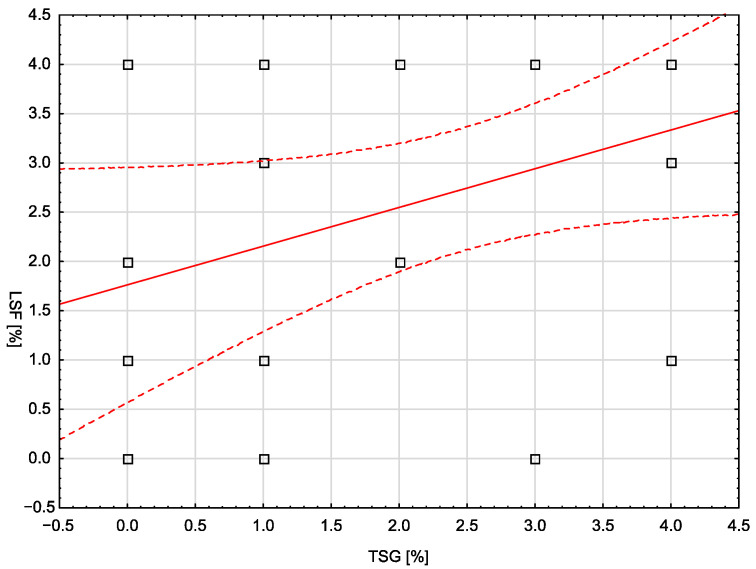
The correlation between TSG and LSF markers in the control group.

In the control group, none of the analyzed demographic, clinical, or laboratory variables correlated statistically significantly with the assessed molecular markers. The only exception was the number of pregnancies, which showed a moderate, positive correlation with the LSF marker (rho = 0.401; *p* = 0.0467). In the study group, a statistically significant, weak, and negative correlation was noted between body weight and the LSF marker (rho = −0.399; *p* = 0.0484). Similarly, a significant correlation (moderate, negative) was noted for BMI and LSF (rho = −0.420; *p* = 0.0365). There was no statistically significant correlation between the TSG and LSF markers in any of the groups. Figure 4 is a scatterplot showing the correlation between TSG and LSF markers in the control group (no statistical significance, *p* = 0.0685).

### 5.3. Assessment of the Diagnostic Usefulness of the Tested Molecular Markers in the Detection of Endometrial Cancer

**Figure 5:**
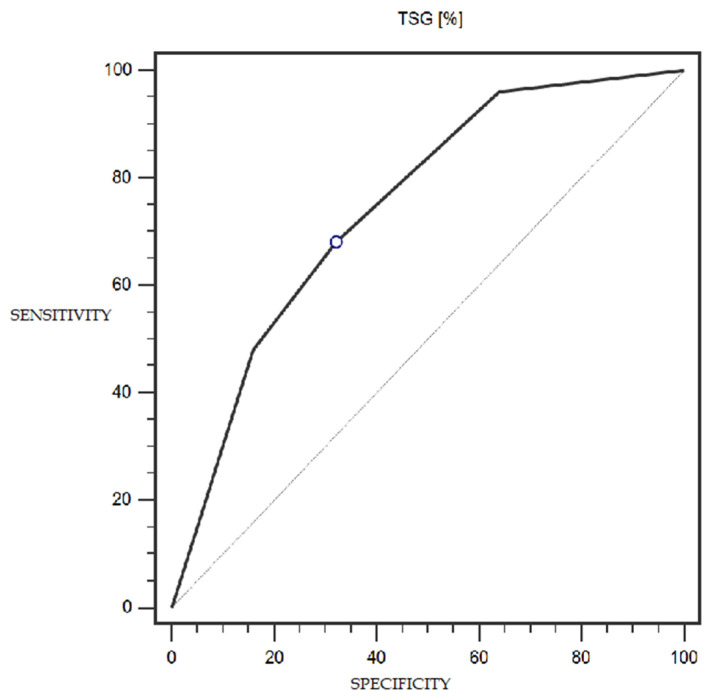
ROC curve representing the diagnostic utility of TSG in detecting endometrial cancer with myometrium infiltration, with positive and negative lymph nodes.

TSG expression was characterized by 68% sensitivity and 68% specificity (with a cut-off point of 25% of cells expressing this marker) in the detection of endometrial cancer (statistically significant result; AUC = 0.75; *p* = 0.0002; Figure 5). The LSF marker did not show statistically significant diagnostic usefulness in detecting endometrial cancer. Detailed data presenting the assessment of the diagnostic usefulness of the tested molecular markers using ROC curves in the detection of endometrial cancer are presented in Table 4.

**Figure 6:**
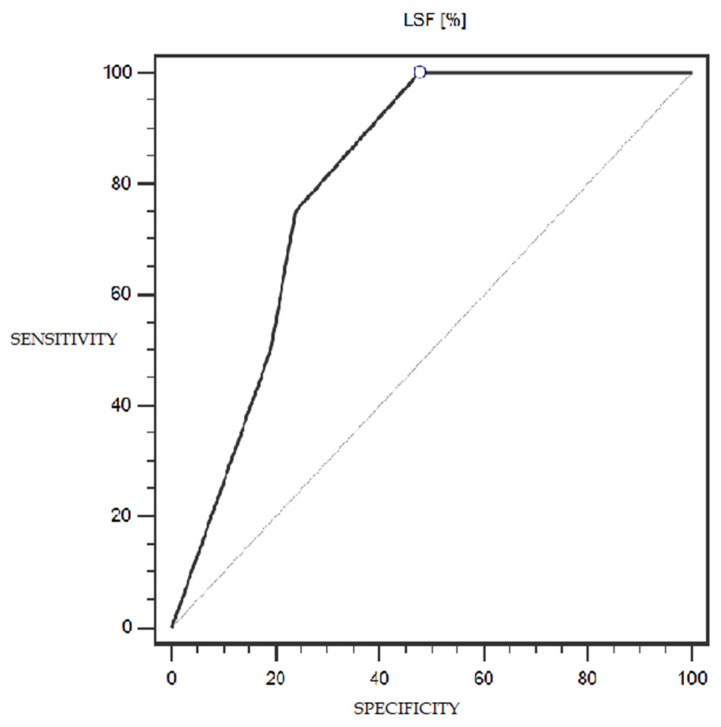
ROC curve representing the diagnostic usefulness of LSF in differentiating patients with more or less advanced disease (IA-IC vs. II-IIIC; according to the FIGO classification).

**Figure 7:**
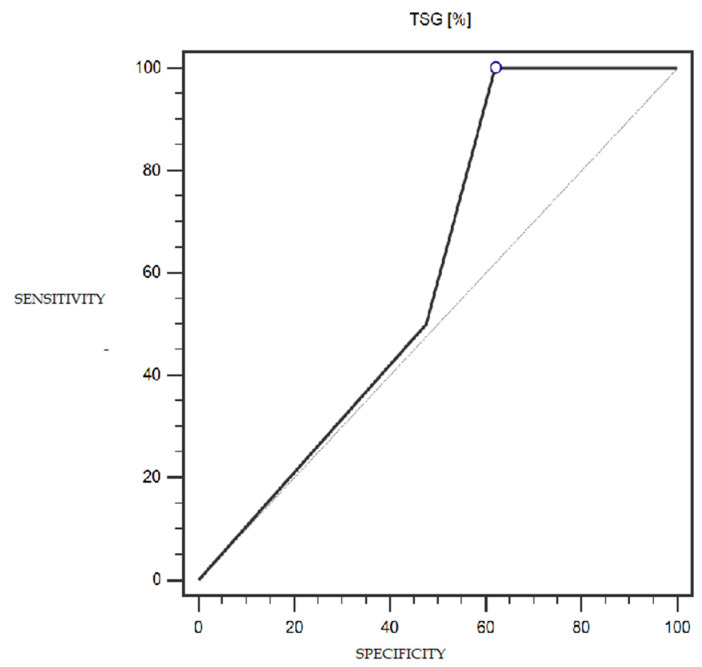
ROC curve representing the diagnostic usefulness of TSG in differentiating patients with more or less advanced disease (IA-IC vs. II-IIIC; according to the FIGO classification).

LSF expression was characterized by 100% sensitivity and 52% specificity (with a cut-off point of 1% of cells expressing this marker) in differentiating patients with more or less advanced disease (IA-IC vs. II-IIIC; according to the FIGO classification) (statistically significant result; AUC = 0.81; *p* = 0.0005; Figure 6). However, the TSG marker did not show statistically significant diagnostic usefulness in differentiating patients with more or less advanced disease (according to the FIGO classification) (Figure 7). Detailed data presenting the assessment of the diagnostic usefulness of the tested molecular markers using ROC curves in distinguishing patients with more or less advanced disease (according to the FIGO classification) are presented in Table 5.

## 6. Discussion

Although endometrial cancer is a disease that is quite well researched in terms of risk factors, intensive research is ongoing on genetic and epigenetic mechanisms that may promote the occurrence of this disease or have a protective effect.

### 6.1. TSG101

In order to determine the role of TSG101 in cancer transformation, Bennett et al. 2001 examined the expression level of this gene’s products in primary ovarian and endometrial gland cancer cell lines [18]. The TSG101 protein was detected in these cell lines without any missing fragments, indicating that they were not characterized by any intragenic deletions within TSG101. Additionally, TSG101 protein levels were compared with deficiencies in molecules important for cell cycle regulation, such as cyclin D1, cyclin E, p16, and p53. Reduced TSG101 gene products were observed in 36% (8/22) of ovarian cancers and 17% of endometrial cancers. The above results suggest that reduced TSG101 expression is associated with carcinogenesis in these tumor groups. Broniarczyk et al. 2014 found that the expression of TSG101 varied depending on the type of cancer, and its expression was not reduced in all cases [10]. Other available data from the literature indicate that it has reduced expression in endometrial and cervical cancers; however, it may be increased in papillary thyroid cancer, breast cancer, gastrointestinal cancers, and even in ovarian cancers [19].

It turns out that the TSG101 gene product is a versatile protein and that its action is complex and multidirectional, which does not seem strange considering its involvement in many cellular processes, including endosomal ordering and exchange, regulation transcription, the cell cycle and proliferation, protein ubiquitination, and cytokinesis. In 2013, Jiang et al. described other types of cancer in which TSG101 expression was increased, such as prostate cancer, lung cancer, and gallbladder cancer [20]. The ability of TSG101 to have both pro- and anti-tumor effects places it among proteins that can regulate the cell cycle in opposing ways depending on the physiological context. The challenge is to determine exactly what effect TSG101 has on carcinogenesis and what specific cell cycle element is key to its action. Moreover, it is worth investigating whether its mechanism of action in each type of cancer is the same, or whether the mechanisms are different depending on the tissue involved.

The matter is further complicated by the fact that the action of the TSG101 gene product may be disturbed not only by somatic mutations but also as a result of epigenetic mechanisms. Changes in the nucleotide sequence in DNA can be both hereditary and caused by external factors. Examples of epigenetic mechanisms include DNA methylation, histone deacetylation, and miRNA expression. Methylation of the promoters of some tumor suppressor genes is responsible for their silencing and may promote carcinogenesis. Similarly, histone deacetylation can lead to the activation of oncogenes. MiRNA molecules are small, 18–20 nucleotide-long, non-coding RNA fragments capable of inhibiting other RNA molecules that may disturb the balance between the expression of oncogenes and tumor suppressor genes. It has been shown that the growth of some types of tumors can be stimulated by epigenetic changes at various stages of carcinogenesis, and xenobiotics capable of interfering with these mechanisms may promote the process of carcinogenesis. However, epigenetic changes are dynamic and can be reversed with specific measures’ inhibitors.

In recent years, methyltransferase and histone deacetylase inhibitors have attracted the attention of researchers and clinicians because it has become widely believed that they may constitute a viable therapeutic option in certain types of tumors. Drugs that inhibit histone methylation or deacetylation are being tested for their ability to reactivate tumor suppressor genes and repress cancer cell growth. Such epigenetic factors may act alone or in cooperation with other drugs. This provides enormous room for maneuver in attempts to expand the therapeutic options that could be offered to cancer patients. TSG101 is one of the genes whose susceptibility to epigenetic modifications could be used in oncological therapies [21,22].

Our study showed that a significantly lower percentage of endometrial cancers had high TSG101 expression compared to the normal control tissue (4% vs 36%; *p* = 0.0312). Furthermore, an attempt to investigate the usefulness of the loss of TSG101 expression as a possible diagnostic marker for endometrial cancer showed 68% sensitivity and 68% specificity (AUC = 0.75; *p* = 0.0002), with a cut-off of 25% of cells expressing this marker.

The above data indicate that TSG101 has a role as a tumor suppressor factor for endometrial cancer and even suggest the possibility of its use as a diagnostic or prognostic marker of this disease. Increasing the expression of this protein by altering its epigenetic control could be used as a therapeutic option. Further studies are planned by our team.

### 6.2. LSF

In samples of colorectal, cervical, and ovarian cancers, LSF expression levels are increased, suggesting that this factor may be involved in tumorigenesis and could be useful as a marker and prognostic factor [23]. There have already been attempts to investigate the utility of LSF in combination with CA-125 (cancer antigen 125) as a biomarker for ovarian cancer [24]. However, the role of LSF in the development of colorectal, cervical, and ovarian cancer remains uncertain, and the molecular mechanisms in which it could participate require further research.

In contrast to the above mechanisms, LSF may play a role in the prevention of melanoma [25]. The level of LSF expression in melanoma is reduced compared to expression in the cells from which pigmented nevi are derived, and its overexpression inhibits the growth of melanoma cells by controlling the transcription of the p21 factor.

LSF binds to the promoter of the DAPK protein kinase gene associated with the programmed cell death system and increases its transcription [26]. DAPK promoter methylation prevents LSF binding. DAPK is a tumor growth suppressor that is silenced in many cancers, suggesting that LSF may actually function as a tumor suppressor in some situations. These studies were performed on twelve lung cancer cell lines and three breast cancer cell lines. LSP also enhances the expression of the gene encoding the ASIC2 ion channel by directly binding to its promoter. ASIC2 channel expression is reduced in all glioma types compared to control samples. Despite the lack of direct evidence, this finding may suggest that LSF acts as a suppressor in the process of glial tumor formation [27].

Many new studies have shown that increasing the amount of non-coding RNA (such as micro-RNA and long non-coding RNA) may be linked to numerous diseases. Circulating RNA (circRNA) is an endogenous, non-coding RNA. This molecule has an unusual covalent structure. Many cancer tissues have been found to express different circRNAs compared to healthy tissues. Endometrial cancers also have differential expression of circRNAs, suggesting that these molecules could be useful as a diagnostic and prognostic marker of this cancer [28].

One study discovered a significant increase in the expression of a circRNA molecule called hsa_circ_0023404 in cervical cancer cells compared to healthy cells. hsa_circ_0023404 was shown to activate the YAP signaling pathway in cervical cancer by promoting LSF expression by *“sponging”* miR-136, leading to the development and progression of cancer [29]. Perhaps circRNA molecules using a similar mechanism play a role in the pathogenesis of endometrial cancer via LSF; however, the levels of LSF expression in cervical cancer and endometrial cancer differ. In our study, LSF expression levels were decreased in endometrial cancer.

A somewhat surprising observation was made when trying to assess the utility of the level of LSF expression on the prognosis of patients with endometrial cancer. With a cut-off point of 1% of cells expressing LSF, this expression was characterized by 100% sensitivity and 52% specificity in differentiating patients with more advanced disease (II-IIIC according to FIGO) from patients with less advanced disease (IA-IC according to FIGO); this result was statistically significant (AUC = 0.81; *p* = 0.0005).

The above observations suggest a protective effect of LSF against the occurrence of endometrial cancer, but, paradoxically, its expression was higher in patients with endometrial cancer at a higher clinical stage. This suggests the possibility of using this marker both in the diagnosis and assessment of prognosis in patients with endometrial cancer. However, it is worth emphasizing the complexity and multi-directionality of biological and clinical effects that may result from abnormal LSF expression.

### 6.3. LSF and TSG101

So far, there are very few works describing the connections of LSF with TSG101, but in 2014, Broniarczyk et al. discovered a binding site for LSF in the promoter of the TSG101 gene, so it seems very likely that both of these factors are co-involved in the regulation of carcinogenesis [10]. Cancers for which a possibility of cooperation between the products of both genes has been mentioned are cervical cancer, lung adenocarcinoma, and breast cancer [10,30,31].

The statistical analysis of the current research revealed a trend toward significance for a correlation between the TSG101 and LSF markers (weak positive correlation, rho = 0.370; *p* = 0.0685). However, despite the correlation not reaching the level of significance, it indicates the possibility of some interdependencies between the factors, especially since the literature contains data confirming the existence of an LSF binding site in the TSG101 promoter [10]. Although literature reports on the relationship between TSG101 and LSF are scarce, and the correlations we found are weak, linking these two markers could be useful in the diagnosis and assessment of prognosis of endometrial cancer, but this issue requires further research.

## 7. Conclusions

1. A much lower level of TSG101 expression was observed in endometrial cancer than in the non-cancerous endometrium, and it may have utility as a diagnostic marker of endometrial cancer.
2. Decreased expression of LSF may be a marker of low-grade endometrial cancer, and increased expression of LSF may indicate progression of the disease and a worse prognosis.
3. The effect of the LSF transcription factor on the regulation of TSG101 expression remains unknown, and this issue requires further research.

## Figures and Tables

**Table 1:** Comparison of demographic, clinical, and molecular variables in the control and study groups.

	*n* (%) ^a^ or Mean ± Standard Deviation Median (Interquartile Range) Min–Max ^b,c^		*p*-Value
	Control Group	Study Group	
**Age (years)**	65 (55.7–70.8)	68 (58.7–74.2)	0.2214
**Body weight (kg)**	73 ± 13.5	73.2 ± 13.3	0.9580
**BMI (kg/m^2^)**	27 ± 4.6	28 ± 4.9	0.4634
**Age at first menstruation (years)**	13 (12–14.5)	14 (13–15)	0.2582
**Pregnancies**	2 (1–3)	2 (1–2)	0.7929
**Births**	2 (1–2)	2 (1–2)	0.7285
**Comorbidities—diabetes**			0.4705
Yes	-	4 (8%)	
No	50 (100%)	46 (92%)	
**Comorbidities—arterial hypertension**			0.0020 *
Yes	4 (8%)	26 (52%)	
No	46 (92%)	24 (48%)	
**TSG**			0.0312 *
**0%**	8 (16%)	24 (48%)	
**<25%**	18 (16%)	10 (20%)	
**25–50%**	6 (12%)	6 (12%)	
**50–75%**	10 (20%)	8 (16%)	
**>75%**	18 (36%)	2 (4%)	
**LSF** **0%** **<25%** **25–50%** **50–75%** **>75%**	8 (16%) 6 (12%) 4 (8%) 6 (12%) 26 (52%)	6 (12%) 16 (32%) 12 (24%) 4 (8%) 12 (24%)	0.1260

^a^ Categorical data, ^b^ linear (continuous) data, ^c^ in the absence of a normal distribution of data in any of the compared groups, medians and interquartile ranges are reported; in other cases, means and standard deviations are reported, * statistically significant.

**Table 2:** Characteristics of the study group.

	*n* (%) ^a^	Normal Data Distribution (Yes/No)
**Clinical diagnosis (according to ICD-10) C54**	50 (100%)	-
**FIGO**		
IA IB II IIIB IIIC	16 (32%) 26 (52%) 2 (4%) 4 (8%) 2 (4%)	No
**Nodal status**		
N−	46 (92%)	No
N+	4 (8%)	
**Histopathological diagnosis**		
Endometrial cancer—glandular type	50 (100%)	-
**Degree of histopathological malignancy**		
G1	14 (28%)	Yes
G2	24 (48%)	
G3	12 (24%)	

^a^ Categorical data.

**Table 3:** Assessment of the risk of developing endometrial cancer depending on the occurrence of specific molecular changes.

		*n* (%)		OR (95% CI) *p*-Value
		Control Group	Study Group	
**TSG**	**0%** **≥1**	8 (16%) 42 (84%)	24 (48%) 26 (52%)	0.21 (0.05–0.78) 0.0197 *
	**0–25%** **>25%**	16 (32%) 34 (68%)	34 (68%) 16 (32%)	0.22 (0.07–0.73) 0.0129 *
	**0–50%** **>50**	22 (44%) 28 (56%)	40 (80%) 10 (20%)	0.20 (0.06–0.69) 0.0113 *
	**0–75** **>75%**	32 (64%) 18 (36%)	48 (96%) 2 (4%)	0.07 (0.01–0.64) 0.0182 *
**LSF**	**0%** **≥1**	8 (16%) 42 (84%)	6 (12%) 44 (88%)	1.40 (0.28–7.00) 0.6845
	**0–25%** **>25%**	14 (28%) 36 (72%)	22 (44%) 28 (56%)	0.49 (0.15–1.61) 0.2416
	**0–50%** **>50**	18 (36%) 32 (64%)	34 (68%) 16 (32%)	0.26 (0.08–0.85) 0.0262 *
	**0–75** **>75%**	24 (48%) 26 (52%)	38 (76%) 12 (24%)	0.29 (0.09–0.97) 0.0454 *

* Statistically significant result.

**Table 4:** Diagnostic usefulness of the tested molecular markers in detecting endometrial cancer.

	Cut-Off Point	Sensitivity (%)	Specificity (%)	AUC (95% CI)	*p*-Value
**TSG (%)**	≤25%	68%	68%	0.75 (0.60–0.86)	0.0002 *
**LSF (%)**	≤25–50%	68%	6%	0.63 (0.48–0.76)	0.0966

* Statistically significant result.

**Table 5:** Diagnostic usefulness of the tested molecular markers in differentiating patients with more or less advanced disease (IA-IC vs. II-IIIC; according to the FIGO classification).

Variable	Cut-Off Point	Sensitivity (%)	Specificity (%)	AUC (95% CI)	*p*-Value
**TSG (%)**	≤1	100%	38%	0.61 (0.39–0.79)	0.3670
**LSF (%)**	>1	100%	52%	0.81 (0.60–0.94)	0.0005 *

* Statistically significant result.

## Data Availability

The data presented in this study are available on request from the corresponding author.

## Abbreviations

AUC	area under the curve
BMI	body mass index
DNA	deoxyribonucleic acid
EIN	endometrial intraepithelial neoplasia
FIGO	French Federation Internationale de Gynécologie et d’Obstetrique (International Federation of Gynecology and Obstetrics)
LSF	late simian virus 40 factor (SV40 late transcription factor (synonym: TFCP2))
OR	odds ratio
PIK3CA	mutation in breast cancer
PTEN	phosphatase and tensin homolog deleted on chromosome ten (protein encoded by the gene suppressor PTEN located on the long arm of chromosome 10)
RNA	ribonucleic acid
RR	relative risk
SV40	simian virus 40 (monkey virus)
TCGA	The Cancer Genome Atlas
TSG101	tumor susceptibility gene 101
WHO	World Health Organization (global health organization)
